# Immunotherapy for Hepatocellular Carcinoma: Current Limits and Prospects

**DOI:** 10.3389/fonc.2021.589680

**Published:** 2021-03-29

**Authors:** Cheng Zhong, Yirun Li, Jing Yang, Shengxi Jin, Guoqiao Chen, Duguang Li, Xiaoxiao Fan, Hui Lin

**Affiliations:** ^1^Department of General Surgery, Sir Run Run Shaw Hospital, School of Medicine, Zhejiang University, Hangzhou, China; ^2^Biomedical Research Center, Sir Run Run Shaw Hospital, School of Medicine, Zhejiang University, Hangzhou, China; ^3^State Key Laboratory of Modern Optical Instrumentations, Centre for Optical and Electromagnetic Research, College of Optical Science and Engineering, International Research Center for Advanced Photonics, Zhejiang University, Hangzhou, China

**Keywords:** immunotherapy, hepatocellular carcinoma, immune tolerance, tumor mutational burden, tumor microenvironment, epigenetic modification, tertiary lymphoid structure

## Abstract

Although many approaches have been used to treat hepatocellular carcinoma (HCC), the clinical benefits remain limited, particularly for late stage HCC. In recent years, studies have focused on immunotherapy for HCC. Immunotherapies have shown promising clinical outcomes in several types of cancers and potential therapeutic effects for advanced HCC. In this review, we summarize the immune tolerance and immunotherapeutic strategies for HCC as well as the main challenges of current therapeutic approaches. We also present alternative strategies for overcoming these limitations.

## Introduction

Liver cancer is the seventh most commonly diagnosed cancer and third leading cause of cancer-related death worldwide. Hepatocellular carcinoma (HCC), the most common form of liver cancer, shows high morbidity and mortality (1). The major clinical risk factor for developing HCC is liver cirrhosis. Chronic infections with hepatitis B virus and hepatitis C virus as well as long-term heavy alcohol consumption are the main causes of cirrhosis development (2).

Surgical resection, liver transplantation, and radiofrequency ablation (RFA) are widely applied in the clinical treatment of early stage HCC (Barcelona Clinic Liver Cancer [BCLC] stage A). For patients with intermediate HCC (BCLC stage B), transarterial chemoembolization is considered as the first-line treatment with a median survival of approximately 40 months (3–5). However, most patients with HCC are first diagnosed in an advanced stage (BCLC stage C). The multi-tyrosine kinase inhibitors sorafenib and regorafenib have been approved by the Food and Drug Administration as first- and second-line treatments for advanced HCC but only increase survival by less than three months (5). Although many treatment approaches have improved the clinical efficacy, patients with HCC suffer from tumor recurrence and show poor survival rates. Thus, novel therapeutic strategies are urgently needed.

Cancer immunotherapy (CIT) has rapidly developed in the past few years and has improved the survival of patients with different tumors. However, only a few patients with specific cancers, such as melanoma or Hodgkin’s lymphoma, exhibit life-altering improvements with CIT. Most patients with solid tumors still respond negatively to immune therapies. In this review, we summarize the immune tolerance and immunotherapeutic strategies for HCC, analyze the limits of current therapeutic approaches, and present alternative strategies which might overcome these limitations.

## Immune Tolerance of HCC

The liver is constantly exposed to non-self proteins derived from nutrients or microbiota, which can trigger immune responses. Many mechanisms protect these harmless antigens from being attacked by the hepatic immune system to maintain homeostasis in the hepatic microenvironment (6). In chronic liver disease, continuous inflammation makes the liver an immunosuppressive microenvironment. Chronic hepatitis B virus and hepatitis C virus infection are the most important risk factors for HCC and are associated with 80% of HCC cases globally (7, 8), providing an immunosuppressive milieu for the initiation and progression of HCC (9). Tumor cells and the specific immune system of HCC constitute an immune-resistant microenvironment, allowing tumor tissue to evade the surveillance of the immune system and protecting the tumor tissue from immune system attack.

### Tumor Cells Promote Immune Tolerance of HCC

Hepatocytes under chronic pressure gain ‘driver’ mutations (10), leading to growth advantages and gradually transforming them into low-grade dysplastic nodules, high-grade dysplastic nodules, early HCC, and finally advanced HCC (11). The progression of tumor cells under the selective pressure of immune system resulting in the emergence of immune-resistant tumor cells with fewer immunogenic or immunosuppressive factors is named as ‘immunoediting’ (12).

Tumor cells show weakened antigenicity. Tumor-associated antigens (TAAs) are antigens that are either only produced by tumor cells or overexpressed in tumors compared to in normal cells. The most studied TAAs are oncofetal antigens and cancer/testis antigens, including alpha fetoprotein (AFP), glypican-3 (GPC-3), New York esophageal squamous cell carcinoma-1, synovial sarcoma X-2, melanoma antigen gene-A, and human telomerase-reverse transcriptase, which can elicit a defensive immune response in the host. In the progression of a chronically inflamed liver and HCC, genetic and epigenetic alterations under pressure from the microenvironment transform tumor cells and deregulate the expression of TAAs. In addition to decreasing TAAs, HCC cells escape immune attack by releasing immunosuppressive cytokines, such as transforming growth factor-β and indoleamine 2, 3-dioxygenase (13, 14).

### Immunosuppressive Cells in HCC

The liver prevents harmless antigens from being attacked by the hepatic immune system and thus maintains homeostasis in the hepatic microenvironment (6). However, long-lasting inflammatory and antigenic stimulation switches the immune system in the liver to an immunosuppressive status, which is exacerbated during the initiation and progression of HCC (15).

#### Repressive T Cells in HCC

The mechanisms of immunological tolerance for T cells in HCC including inactivation or deletion of effector T cells, mainly refers to CD8^+^ T cells as well as priming and expansion of regulatory T cells (T_reg_ cells). The presence of tumor-infiltrating lymphocytes (TILs) is associated with a good prognosis and improved overall survival in HCC (16). Cytotoxic infiltrating CD8^+^ T cells are the major cell type functioning to kill tumor cells. However, persistent exposure to antigens stimulates effector CD8^+^ T cells to differentiate into exhausted CD8^+^ T cells (17). Exhausted CD8^+^ T cells were originally characterized by down-regulated expression of interferon gamma (IFN-γ) during chronic inflammation. Poor expression of tumor necrosis factor-β and interleukin-2 (IL-2) in exhausted CD8^+^ T cells is also observed, resulting in impaired cytotoxic function (18). In addition to the loss of effector function, exhausted CD8^+^ T cells express inhibitory receptors (IRs), such as programmed cell death 1 (PD-1), lymphocyte-activation gene 3, T cell immunoglobulin domain and mucin domain-containing protein 3, and cytotoxic T lymphocyte-associated antigen (CTLA)-4 (19–21). IRs are negative regulatory pathways that prevent the immune system from attacking cells indiscriminately. However, in the tumor immune system, IRs protect tumor cells from immune system attack. Persistent and elevated expression of these IRs has been observed in HCC (22, 23).

T_reg_ cells are a subpopulation of T cells that modulate the immune system and play an immune suppressive role in immune tolerance in cancer. Depletion of T_reg_ cells results in severe autoimmunity and allergies (24–26). In HCC, accumulation of intra-tumoral T_reg_ cells correlates with tumor progression and poor prognosis (27, 28). T_reg_ cell depletion can also activate an effective immune response in tumor models in animals (29, 30). T_reg_ cells express the CD4, CD25, and Foxp3 biomarkers. Foxp3 is a key regulatory gene in the development of T_reg_ cells (31). The transcription factor Foxp3 has been proposed to regulate the expression levels of immune-suppressive molecules in T_reg_ cells. Ectopic expression of Foxp3 confers Treg-like suppressive function to CD4^+^CD25^-^ T cells (32), and various molecules encoded by Foxp3-controlled genes are associated with immune suppression (33).

#### Myeloid Cells in HCC

There are two types of myeloid cells; marrow-derived suppressor cells (MDSCs) and tumor associated-macrophages (TAMs), which play important roles in the tumor microenvironment. MDSCs are a population of immature myeloid cells with strong immunosuppressive functions and can promote tumoral angiogenesis. MDSCs can differentiate into macrophages, granulocytes, and dendritic cells (DCs) (34). However, in the hypoxic microenvironment of HCC, tumor cells express ectonucleoside triphosphate diphosphohydrolase 2, which can convert extracellular ATP to 5′-AMP and thus prevent the differentiation of MDSCs (35). Arginine is an essential amino acid for the proliferation of CD4^+^ and CD8^+^ T cells. MDSCs suppress T-cell proliferation *via* increased arginase activity, leading to arginine depletion (36). MDSCs also exert an immunosuppressive effect by inducing the differentiation of CD4^+^ T cells into T_reg_ cells (36).

TAMs are also immunosuppressive myeloid cells. Macrophages can differentiate *via* two routes, known as macrophage polarization. Classically activated macrophages (M1) produce high levels of IL-12 and low levels of IL-10 and promote tumor initiation, whereas alternatively activated macrophages (M2) are characterized by low IL-12 and high IL-10 production and promote tumor progression. The microenvironment of HCC stimulates macrophages towards M2 polarization, which are named as TAMs (37). A previous study reported that macrophages in the early stage of HCC express high levels of major histocompatibility complex (MHC)-class II and cytokines, such as IL-1β, IL-6, IL-12, and inducible nitric oxide synthase, which suppress tumor progression. However, in advanced HCC, macrophages express M2-like molecules, including macrophage mannose receptor c1, arginase, IL-10, and transforming growth factor-β and low levels of MHC-class II, which promote tumor progression (38).

TAMs promote tumor progression through angiogenesis, tumor cell invasion, and metastasis (39). Infiltrating TAMs contribute to poor prognosis in HCC, and *in vivo* and *in vitro* experiments have shown that TAMs in HCC enhance tumor invasion by producing C-C motif chemokine 22 (40). Another study showed that TREM-1^+^ TAMs in HCC induce immunosuppression by recruiting C-C chemokine receptor type 6-positive T_reg_ cells, releasing CCL20 and producing the immune checkpoint molecule PD-L1 which may endow HCC with anti-PD-L1 therapy resistance (41). Transforming growth factor-β in the HCC environment can promote TAMs to produce T-cell immunoglobulin- and mucin-domain-containing molecule-3, which can promote bone marrow-derived macrophages and peripheral monocytes to differentiate into TAMs (42). After co-culture with tumor cells, TAMs promoted the expansion of CD44^+^ HCC stem cells by producing IL-6 and signaling *via* STAT3 (43). The CCR2^+^ macrophage subset has pro-angiogenic properties in HCC, and inhibition of CCR2^+^ TAMs in the fibrosis-HCC model significantly suppress angiogenic activities (44).

#### Hepatic Stellate Cells in HCC

Hepatic stellate cells (HSCs) are the main producers of extracellular matrix in the liver. In liver fibrosis, HSCs are activated towards a myofibroblast-like phenotype and play a key role in fibrogenesis (45). Activated HSCs produce extracellular matrix, cytokines, and growth factors to create a tumor-favoring environment in HCC (46). Activated HSCs in HCC suppress the antitumor immune response by depleting effector T cells and promoting the accumulation of immunosuppressive cells. HSCs can induce apoptosis of activated T cells through PD-L1 signaling (47, 48). Activated HSCs can convert mature peripheral blood monocytes into MDSCs (49). In murine models, HSCs can present antigens to naïve CD4+ T cells and transform activated naïve CD4+ T cells into Foxp3+ Treg cells by producing retinoic acid (50).

#### Liver Sinusoidal Endothelial Cells in HCC

Liver sinusoidal endothelial cells (LSECs) form a bed in the liver and receive blood from both the hepatic artery and portal veins in the hepatic parenchyma. In addition to functioning as vascular channels, LSECs play a role in the immune system. LSECs function in both pathogen recognition and antigen presentation. LSECs can cross-present antigens to CD8^+^ T cells by taking up, processing, and transferring antigens to MHC class I. The presentation of antigens produces a tolerogenic response in naïve CD8^+^ T cells by upregulating PD-L1 on the surface of LSECs, which bind to the PD-1 receptor expressed on naïve CD8^+^ T cells (51). LSECs also present antigens to the MHC class II complex to activate CD4^+^ T cells. However, because of the lack of co-stimulatory molecules, LSECs drive naïve CD4^+^ T cells to develop into T_reg_ cells rather than into T helper cells (52). LSECs express various receptors for angiogenic factors including vascular endothelial growth factor receptors 1 and 2, Tie‑2 (angiopoietin‑1 receptor), and platelet-derived growth factor receptor. The interaction between these receptors and their ligands promotes the proliferation of LSECs and angiogenesis (53, 54).

## Immunotherapy for HCC

Immunotherapy for cancer mainly involves three approaches: vaccines, adoptive cell transfer (ACT), and immune checkpoint inhibitors (ICIs) (**Table 1**). Vaccines or ACT with genetically modified T cells target specific antigens. ICI inhibits the suppressive regulators of T cells and stimulates already present antitumor immune responses to kill tumor cells.

**Table 1:** Clinical trials of immunotherapy for HCC.

Therapy approaches	Phase	Agents or approaches	Population	Endpoints	Relevant finding	Reference
Vaccine	I	AFP-derived peptides Vaccines	15 patients with advanced HCC	P: safety S: immune response	No AE; CR, 1; PR, 8.	(55)
Vaccine	I	GPC3 peptide vaccine	33 patients with advanced HCC	P: safety S: immune response	No AE; PR, 1; SD, 19; GPC3-specific CTL response in 30 patients; MST in patients with CTL frequencies ≥ 50 (N=15), 12.2 months; MST in patients with CTL frequencies < 50 (N = 18), 8.5 months	(56)
Vaccine	II	cyclophosphamide and a telomerase peptide (GV1001) vaccine	40 patients with advanced HCC	P: tumor response S: TTP, TTSP, PFS, OS, safety and immune responses.	SD: 17; TTP: 57 days; TTSP: 358 days; GV1001 treatment result in a decrease of regulatory T cells.	(57)
Vaccine	I/IIa	DC vaccine	12 patients	AE, TTP and RFS	AE, no grade 3 or 4 AE; TTP, 38.4 months; the 1-, 2-, and 5-year RFS, 75%, 69% and 41.7% respectively.	(58)
CIKs	III	CIKs therapy after curative treatment (control, curative treatment without CIKs therapy)	230 patients with HCC	P: RFS S: OS, cancer-specific survival, and safety	The median time of RFS (44 months vs 30 months); AEs, (62% vs 41%), no difference in serious AEs, (7.8% vs 3.5%).	(59)
TILs	I	TILs therapy after tumor section	15 patients with HCC	P: safety	Alive 15, Tumour recurrence: 3.	(60)
ICB	I/II	Nivolumab	48 patients with advanced HCC	P: safety and tolerability for the escalation phase and RR	Grade 3/4 treatment-related adverse events, 12 (25%); treatment-related serious adverse events, 6%; RR, 20% in the dose-expansion phase; RR, 15% in the dose-escalation phase.	(61)
ICB	II	Pembrolizumab	28 patients with advanced HCC	Safety, immune response, PFS and OS	CR, 1; PR, 8; SD,4; the median PFS, 4.5 months; the median OS, 13 months;	(62)
ICB and Antiangiogenic therapy	Ib	Atezolizumab and bevacizumab vs. Atezolizumab	223 patients with unresectable hepatocellular carcinoma	Safety and PFS	PFS (5.6 months vs 3.4 months); serious AE (12% vs 3%)	(63)
ICB and Antiangiogenic therapy	III	Atezolizumab and bevacizumab vs. Sorafenib	501 patients with unresectable hepatocellular carcinoma	OS, PFS and AE	OS at 12 months (67.2% vs 54.6%); PFS (6.8 months vs 4.3 months); Grade 3 or 4 AEs (56.5% vs 55.1%)	(64)
ICB and Ablation	I/II	Tremelimumab with RFA or chemoablation	32 patients with HCC	PR, PFS and OS	PR, 26.3%; PFS at 6 months and 12 months, 57.1% and 33.1%	(65)
ICB and Cytokines	I	mogamulizumab (anti-CCR4 antibody) and nivolumab	15 patients with HCC	Safety, PFS, OS and PR	No AEs; PFS, 3.8 months; OS: 11.3 months; PR, 27%.	(66)

HCC, hepatocellular carcinoma; AFP, alpha fetoprotein; P, primary endpoint; S, secondary endpoint; AE, adverse effect; CR, complete response; PR, partial response; GPC-3, carcinoembryonic antigen glypican-3; SD, stable disease; CTL, cytotoxic T lymphocyte; OS, overall survival; MST, median survival time; PFS, progression-free survival; RFS, recurrence-free survival; TTP, time to progression; TTSP, time to symptomatic progression; RR, response rate; CIK, cytokine-induced killer cell; TIL, tumor-infiltrating lymphocyte; ICB, immune checkpoint blockade; RFA, radiofrequency ablation; CCR4, CC chemokine receptor 4.

### Vaccines

Vaccines have been widely used to prevent various diseases by providing active acquired immunity. Clinical studies using neoantigen peptide, mRNA, or DC vaccines in patients with melanoma have achieved promising results (55, 58, 67, 68). This antigen-based immunotherapy has also been tested for other tumors, such as ovarian cancer, breast cancer (56), and small-cell lung cancer (57). TAAs released from tumor lysates are considered to be optimal vaccines to activate immune response, but the low representation of the TAAs with high immunogenicity limits the clinical effect (69, 70).

Some vaccines are being evaluated for treating HCC. In a phase I trial, administration of AFP-derived peptides as an anti-tumor vaccine was explored in 15 patients with advanced HCC. The results demonstrated that the vaccine was safe and effective. The peptides stimulated the immune system to produce peptide-specific T-cell receptors (TCRs), with one patient showing a complete response and eight patients exhibiting slowing of tumor progression (71). In a phase I trial, a carcinoembryonic antigen glypican-3 (GPC3) peptide vaccine was explored for treating advanced HCC, with 30 of 31 patients (91%) showing a peptide-specific CTL response. For the clinical response among 33 patients, one patient showed a partial response and 19 had stable disease for 2 months (72). A telomerase peptide was also explored as a vaccine target for the treatment of advanced HCC in a phase II trial. No patients showed a complete or partial response, and 17 patients (45.9%) had stable disease for six months (73). Currently, a multi-epitope multi-HLA peptide vaccine is being evaluated in a phase I/II clinical trial for 40 patients with early and intermediate stages of HCC (HepaVac-101-NCT03203005). The results are extremely expected. In addition, lack of high immunogenic vaccines restricts the development of vaccine. A new prediction algorithm is needed for the identification of neoantigens with high immunogenicity, which may have unique homology compared with any human self-antigen and induce vigorous immune response (74–76).

### Adoptive Cell Transfer

Patients receiving ACT therapy are directly treated with autologous natural or engineered anti-tumor T cells (77). The transferred cells can divide into three types, including cytokine-induced killer (CIK) cells, TILs, and genetically modified T cells. CIK cells and TILs can enhance the overall immune response by increasing the number of immune cells, whereas the genetically modified T cells target specific antigens.

#### Cytokine-Induced Killer Cells

CIK cells are a mixture of cytotoxic T cells and natural killer (NK) cells separated from peripheral blood mononuclear cells and are cultured *in vitro* under treatment with cytokines such as IFN-γ, anti-CD3 antibody and IL-2 to promote their proliferation and anti-tumor activities (78). Reinfusion of expanded and activated CIK cells either alone or as a combined therapeutic strategy has been widely studied to suppress tumor progression, with some impressive results observed in metastatic colorectal cancer, myeloid leukemia, and renal cell carcinoma (79–81). Some studies investigated the efficiency of ACT with CIK cells for HCC treatment. In a Korean phase III clinical trial, CIK cells, including CD3^+^/CD56^+^ cells, CD3^-^/CD56^+^ NK cells, and CD3^+^/CD56^-^ cytotoxic T cells (82, 83), were used as an adjuvant treatment for 230 patients with HCC who had been pre-treated with other curative therapies (surgical resection, RFA, or percutaneous ethanol injection). The results showed that the adjuvant immunotherapy group with activated CIK cells had increased overall and recurrence-free survival compared with the control group without adjuvant therapy (median time of recurrence-free survival: 44 vs 30 months) (59).

#### Tumor-Infiltrating Lymphocytes

The presence of TILs in tumors is associated with good prognosis (60, 84). TILs are obtained from surgical tumor specimens and then cultured *in vitro* with sequential treatment with IL-2 for expansion and anti-CD3 antibody for activation. These proliferative and activated TILs are then transferred back into patients. ACT with TILs has been studied for the treatment of metastatic human papillomavirus-associated carcinomas, with clinical responses occurring in 5 of 18 (28%) patients in the cervical cancer group and 2 of 11 (18%) patients in the non-cervical cancer group (85). A phase I clinical trial confirmed the safety of ACT using TILs in patients with HCC: The toxicity and immune response of therapy with autologous TIL is being tested in an ongoing phase I clinical trial of patients with advanced HCC (ClinicalTrials.gov number: NCT01462903) (86).

#### Genetically Modified T Cells

Heterodimeric antibody receptors expressed on the surface of T cells are known to be tumor antigen-specific TCRs that recognize the antigenic peptide-MHC complex. The gene sequence of TCRs that recognize specific TAAs can be analyzed and introduced into autologous T cells by retroviral or lentiviral vectors (87). These proliferative and activated autologous modified TCR-expressing T cells are reinfused into patients. In response to tumor cells, the cells express the target antigen, leading to effective antitumor activity by releasing cytokines such as IFN-γ, granulocyte macrophage colony-stimulating factor, and tumor necrosis factor alpha-α and directly killing tumor cells (88). An AFP TCR with optimal affinity, function, and safety is being evaluated for its clinical efficacy in an early phase clinical trial (ClinicalTrials.gov number: NCT03971747) (89).

Despite the powerful anti-tumor function of immunotherapies based on the interaction between peptide-MHC molecules and TCRs, tumor cells can escape immune surveillance by down-regulating peptide-MHC complex expression (90). ACT with T cells engineered to express a chimeric antigen receptor (CAR) are not limited by the presentation of MHC molecules on the tumor cell surface. CAR can recognize a defined TAA on the surface of tumor cells *via* the single chain variable fragment region, which is constructed from the variable heavy and variable light sequences of a monoclonal antibody specific for TAAs. Activation signals are transferred into cells by activating the transmembrane adaptor signaling protein CD3ζ and one or more co-stimulatory molecules (CD28, CD137, or OX40). The mechanism of CAR therapy causes tumor variants, which can escape the immune surveillance through deficiencies in antigen presentation, to remain susceptible to CAR therapy (87). Other biomarkers have also been considered as targets for CAR T cell therapy. AFP is a well-known biomarker for HCC, and CAR T-cell therapy targeting the AFP-MHC complex showed robust antitumor activity in AFP-CAR T cells in a mouse xenograft model of liver cancer (91). Another attractive liver cancer-specific target is GPC3 because of its high expression in HCC but low expression in normal tissues (92). GPC3-CAR T cells efficiently eradicated GPC3+ HCC cells rather than GPC3- HCC cells. This approach showed high treatment efficiency in an HCC xenograft model with high levels of GPC3 expression and low treatment efficiency in HCC xenografts with low GPC3 expression (93). Another ACT study of GPC3-CAR T cell transfer into patient-derived HCC xenografts also revealed suppression of tumor cell growth (94).

### Immune Checkpoint Inhibitors

The human immune system is in an equilibrium state. Immune checkpoints regulate immune function by suppressing immune activity, interrupting the immune response to avoid overactivation of T cells, and protecting tissues from damage caused by an excessive immune response. Immune checkpoints in tumor tissues promote immune evasion. Most studies of immune checkpoints focused on cytotoxic T-lymphocyte antigen-4 and PD-1 with corresponding PD-L1 ligands. The ICI approach results in nonspecific immune stimulation by targeting negative regulators of T cell signaling pathways.

#### CTLA-4 Inhibitors

The hepatic microenvironment contains a large number of DCs, which are the major antigen-presenting cells in the liver (95). In a normal hepatic microenvironment, DCs take up foreign peptides and present them to T cells *via* the TCR (signal 1). In addition to signal 1, activation of T cells requires co-stimulatory molecules from DCs. After stimulation by the peptide-MHC complex, DCs present CD80 and CD86 to T cells and bind to the CD28 receptor on the surface of T cells (signal 2) and further promote maturation, proliferation, activation, and survival of naïve T cells. Signal 2 prevents the recognition of self-antigens, whereas the absence of such a signal leads to T cell anergy. Upon activation, T cells induce CTLA-4 to competitively bind to CD80 and CD86 with higher affinity than CD28, to prevent an excessive immune reaction (96). CTLA-4 inhibitors prevent CTLA-4 from binding to CD80 and CD86, thereby initiating signal 2, which can activate specific T cells in lymphoid organs and promote their migration into the tumor (97).

Two anti-CTLA-4 monoclonal antibodies, ipilimumab and tremelimumab, are being evaluated in clinical trials for the treatment of other tumors. Studies have shown that CTLA-4 inhibitors deplete T_reg_ cells in the tumor, leading to enhanced effector function of antigen-specific T cells in the tumor. Patients with melanoma and administered ipilimumab exhibit T_reg_ cells depletion (98, 99). However, the clinical data associated with application of CTLA-4 inhibitors alone for advanced HCC are limited. A clinical trial of tremelimumab in patients with HCC and chronic hepatitis C revealed a partial response rate of 17.6% and disease control rate of 76.4% (ClinicalTrials.gov number: NCT01008358) (100).

#### PD-1/PD-L1 Inhibitors

The cell surface receptor PD-1 is expressed on activated T, B, and NK cells and binds PD-L1 and PD-L2 ligands to convey co-inhibitory signals to the TCR. The PD-1 signal terminates immune responses appropriately and maintains self-tolerance by causing apoptosis of antigen‐specific T cells, attenuation of TCR‐mediated activation, and proliferation of T cells (101). The PD-1 signal mediates the function of T_reg_ cells by promoting their differentiation and proliferation (102). These ligands are expressed on leukocytes and tumor cells (103). PD-L1 binding results in phosphorylation of PD-1, inhibiting T cell proliferation and cytokine releasing through SHP2. SHP2 dephosphorylation results in dephosphorylation of key TCR signaling components, most notably CD28 and the ZAP70/CD3zeta signalosome (104, 105). When chronically exposed to antigens, overexpression of PD-1 in T cell induces their exhaustion.

Anti-PD-1 monoclonal antibodies, such as nivolumab and pembrolizumab, and anti-PD-L1 monoclonal antibodies, such as durvalumab and atezolizumab, have been approved for several hematologic and solid malignancies. Many clinical trials for HCC are underway. In a phase I/II of escalation trial, safety was evaluated in 48 patients treated with nivolumab, with grade ≥3 adverse events observed in 31% of patients (15 of 48), which was considered to be a manageable safety profile (61). A phase II study of the efficacy of pembrolizumab in 28 patients showed that one patient achieved a complete response and eight patients achieved partial responses (62).

## Limits of Current Immunotherapies

**Figure 1:**
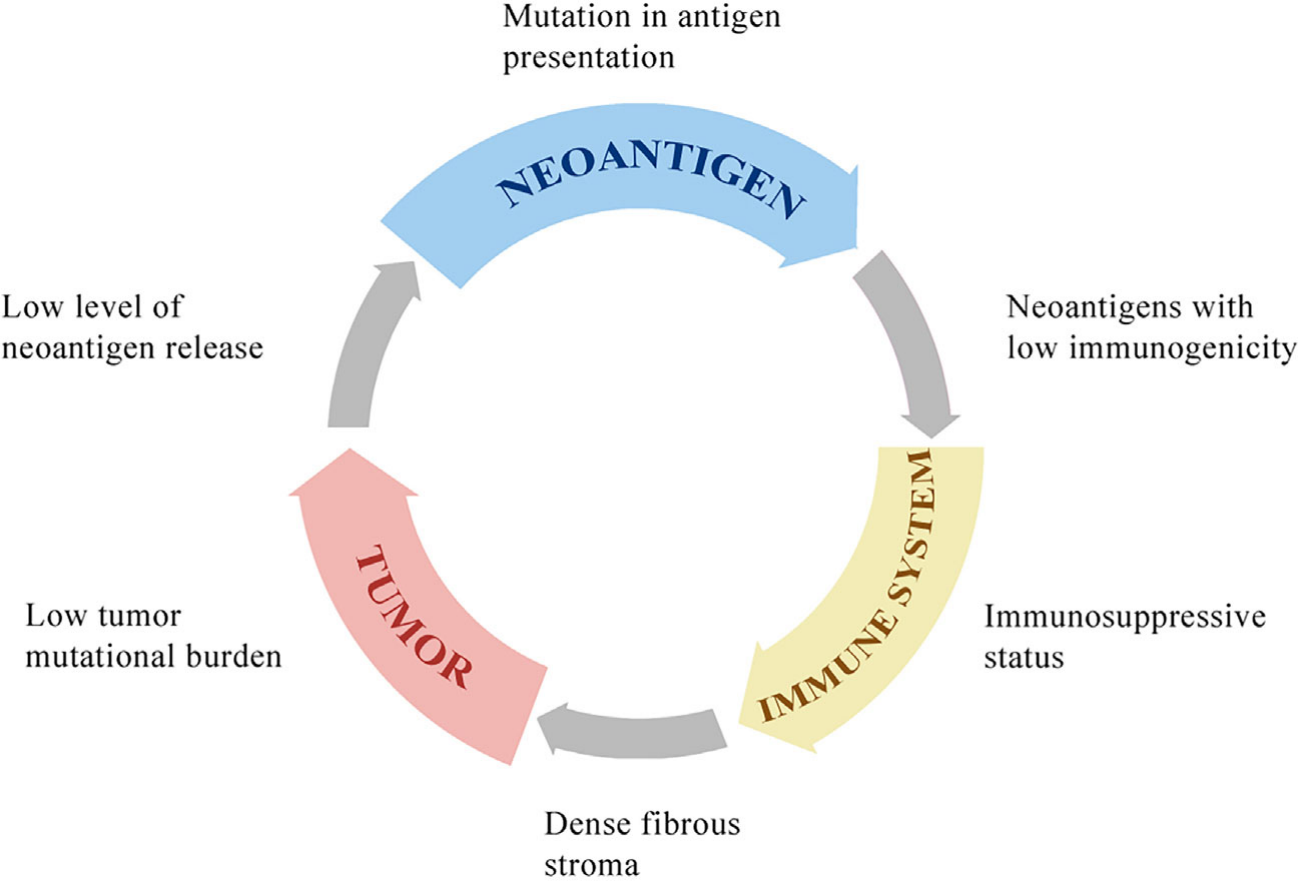
The potential mechanisms of resistance to immunotherapies. HCC with low tumor mutational burden releases few neoantigens. The mutation in antigen presentation pathways also inhibits tumor-specific peptide presentation. Most of these neoantigens cannot drive effective anti-tumor immunity because of low immunogenicity. The immune system in the tumor microenvironment is under immunosuppressive status, with few effector CD8^+^ T cells, many regulatory CD4^+^ T cells, and other immunosuppressive cells, which is associated with poor clinical response to immunotherapy. The dense fibrous stroma around tumor islets inhibits immune cells’ access to the tumor.

Although immunotherapy has shown promising clinical outcomes in some tumors, including melanoma, non‐small cell lung carcinoma, and urothelial carcinoma, the application in HCC faces some limitations (**Figure 1**) (106–108).

### Tumor Mutational Burden

**Figure 2:**
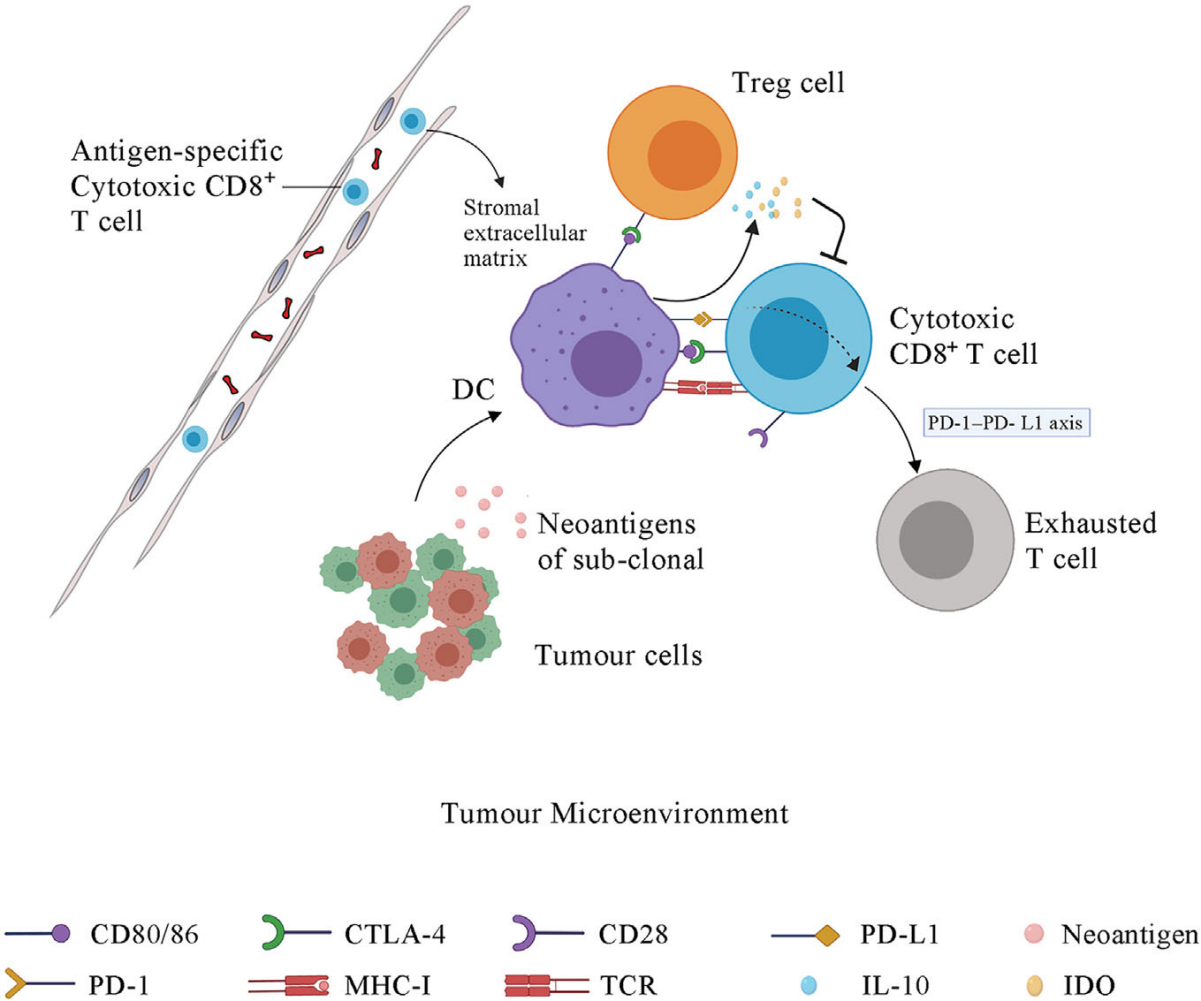
The immune response in tumor microenvironment and the function of immune checkpoints. Some sub-clonal tumor cells release neoantigens while others do not, contributing to the immune response to only part of tumor cells and thus leading to the failure of tumor immunotherapy. Upon antigen recognition, DCs present the antigen-MHC molecules, bind to the TCR on T cell membrane and stimulate the proliferation and activation of CD4^+^ T cells and CD8^+^ T cells in lymph node. Then the antigen-specific cytotoxic T cells migrate to tumor microenvironment *via* blood system. The stromal extracellular matrix in tumor may prevents T cell infiltration. CTLA-4, which is the membrane receptor of activated T cells, outcompetes CD28 for binding to the CD80/86 expressed on the DC membrane, further inhibiting the signal 2, which is essential for the maturation, proliferation, activation and survival of T cells. The interaction of PD-1 and PD-L1 promotes the differentiation and proliferation of Treg cells and induces the cytotoxic CD8^+^ T cells into an exhausted state. DCs under the influence of CLTA-4 signal and PD-1 signal release some immunosuppressive molecules, such as IL-10 and IDO, which suppress T cells activation. IDO, indoleamine 2,3-dioxygenase; PD-1, programmed cell death protein 1; PD-L1, programmed cell death 1 ligand 1; CTLA-4, cytotoxic T-lymphocyte protein 4; DC, dendritic cell; Treg cell, regulatory T cell.

Immunotherapies are ineffective for HCC because of the low tumor mutational burden of HCC compared to that of melanoma or non‐small cell lung carcinoma (109). Neoantigens are tumor-specific peptides that result from somatic mutations in cancer cells. A larger number of somatic mutations is associated with higher levels of neoantigens, and the tumor mutational burden is used to evaluate somatic mutations in cancer to give a useful estimation of the tumor neoantigen load (110, 111). Neoantigens are newly expressed antigens on the surface of tumor cells and can be recognized and presented to T cells to result in adaptive immune response activation. However, even in tumors with a high tumor mutational burden, such as those with deficiencies in DNA damage repair pathways resulting in the accumulation of DNA mutations, a high mutational load is not related to high levels of neoantigens. In fact, only a minority of mutations generates peptides that bind to MHC molecules and present on the surface of tumor cells, and fewer can be recognized by T cells (67, 112). The antigen presentation pathway in tumor cells can be inhibited by mutations in antigen presentation genes. For example, in metastatic melanomas, the loss of b2-microglobulin may result in defects in antigen presentation and escape from immune recognition (113). Not all neoantigens presented on the surface of tumor cells can drive effective antitumor immunity. A study reported that neoantigens could be expressed on either all tumor cells (clonal) or a subpopulation of tumor cells (subclonal). Tumors with a high load of clonal neoantigens show an excellent response to ICI therapy, whereas tumors with a high load of subclonal neoantigens evade immunotherapy (114) (**Figure 2**).

### Tumor Microenvironment

The tumor microenvironment (TME) contains many components, including bone marrow inflammatory cells, lymphocytes, blood vessels, fibroblastic cells, and the extracellular-derived matrix composed of collagen and proteoglycans. The clinical efficacy of ICI depends on three tumor immune status characteristics. First, antigen-specific CD8^+^ T cells must be present within the TME. Second, the resident immune cell populations in the TME must be polarized towards an immune permissive state. Third, tumor cells must have MHC class I-mediated antigen presentation and PD-1 signaling as the dominant mechanism of immune tolerance. A tumor with these characteristics is vulnerable to ICIs and named as an immune “hot” tumor. Immune “cold” tumors lack these characteristics and are associated with poor clinical response to ICI therapy (115). The absence of CD8^+^ T cells in the TME in several tumor types has been associated with poor clinical outcomes of ICI therapy (116–118). A study of the stroma of human lung tumors showed that the stromal extracellular matrix influences the migration and positioning of T cells (119) (**Figure 2**).

## Efforts to Enhance Immunotherapy for HCC

Although the immunotherapy approaches discussed previously have achieved impressive clinical efficacy in other tumors, they have failed to benefit patients with advanced HCC. The limitations of these approaches are discussed above. Here, we summarize some potential combinatorial strategies for enhancing the effects of immunotherapy for HCC.

### Epigenetic Modulation

Epigenetic modification plays an important role in tumor progression, causing transcriptional aberrations in gene expression and immune function changes, which may result in a favorable TME (120). In contrast, epigenetic therapy has the potential to enhance immunotherapy for HCC by converting an immune “cold” tumor into an immune “hot” tumor (121). Epigenetic therapy can promote the expression of immunogenic antigens on the tumor surface such as cancer testis antigens (122–125). Cancer testis antigens are a group of proteins expressed on male germ cells but not in healthy adult somatic tissues and can serve as target antigens for antitumor immunotherapy (126, 127).

**Figure 3:**
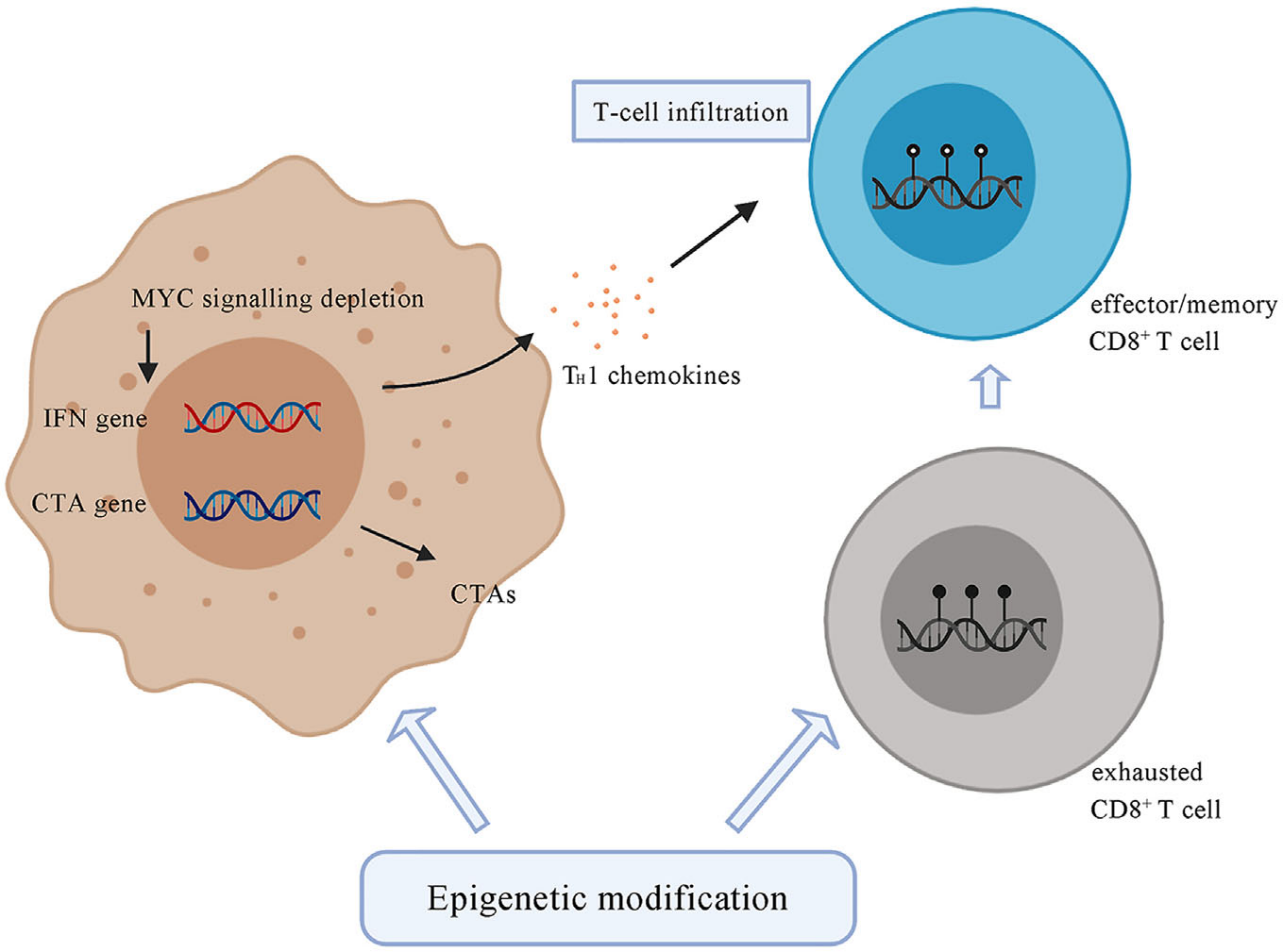
Tumor cells under the treatment of epigenetic drugs upregulate the expression of CTAs, such as NY-ESO-1 and LAGE. Epigenetic modification contributes to the depletion of MYC signalling, activates type I interferon signalling and potentiates the recruitment of T cells. Epigenetic agents can modulate the state of CD8^+^ T cells by transforming exhausted CD8^+^ T cells, which are characterized by a series of changes in effector genes associated with alterations in methylation, into effector or memory CD8^+^ T cells. CTA, cancer testis antigens; NY-ESO-1, New York Esophageal Squamous Cell Carcinoma-1; LAGE, L antigen family member.

Epigenetic modification can regulate the composition of immune cell populations. Methylation of DNA represses genes related to effector function, proliferation, metabolic activity, and tissue homing of exhausted T cells. Chronic antigen stimulation drives CD8^+^ effector T cells towards the exhausted phenotype, which is characterized by a series of changes in gene expression associated with alterations in methylation, leading to increased PD-1 expression and decreased CXCR3 expression (128). *De novo* DNA methylation is essential for establishing exhaustion in T cells, whereas treatment with ICI contributes to rejuvenation of exhausted T cells (128) (**Figure 3**). Azacitidine and histone deacetylase inhibitors have been shown to suppress MYC signaling, activate interferon responsiveness, and potentiate the recruitment of T cells in mouse models of non‐small cell lung carcinoma (129). An EZH2 inhibitor (DZNep) and DNMT1 c (5-azacytidine) can augment anti-PD-L1 immunotherapy for HCC by increasing the release of the chemokines CXCL9 and CXCL10, which stimulate T cell trafficking into the TME. This combination therapy strategy can also upregulate the expression of cancer testis antigens New York esophageal squamous cell carcinoma-1 and L antigen family member, which are normally expressed at low levels, as neoantigens to stimulate the adaptive immune response (130). The potential therapeutic strategy combined with epigenetic modulation has emerged in recent years and is promising for treating HCC.

### Antiangiogenic Therapy

The hypoxia microenvironment stimulates tumor angiogenesis and promotes HCC development. Drugs target angiogenic pathways, including vascular endothelial growth factor (VEGF), are approved for the treatment of advanced HCC (131). Anti-VEGF therapy are widely used in HCC treatment (132). Sorafenib, a tyrosine kinase inhibitor (TKI), can disturb VEGF signaling pathway and approved for HCC treatment (133, 134). Despite survival benefits observed, the high rate of acquired resistance to sorafenib limits its use for advanced HCC treatment.

Despite of high rate of resistance to anti-VEGF drugs for HCC patients, some studies have reported that these drugs can enhance immune response. Drugs targeting VEGF-A/VEGFR-2 axis inhibited T_reg_ cells accumulation in colorectal cancer (135). A VEGFR-2 inhibitor (DC101) promoted tumor-specific CD8^+^ T cells infiltration (136). These findings promote the combined strategies of anti-VEGF drugs and ICBs for HCC treatment. Bevacizumab is an anti-VEGF agent approved to treat metastatic colorectal cancer, glioblastoma, renal cell cancer and cervical cancer (137–139). However, the clinical efficacy of Bevacizumab for HCC treatment was less, with 13% response rates in a phase II study (140). Recent studies focus on the combination of anti-VEGF therapy and immunotherapy. In an open-label, multicenter, multiarm, phase Ib study, atezolizumab plus bevacizumab shows optional results, with longer progression-free survival compare with atezolizumab alone for patients with unresectable HCC (63). A similar result is showed in another clinical trial, the combination strategy of atezolizumab and bevacizumab for HCC treatment showed better overall and progression-free survival outcomes than sorafenib in 501 patients with unresectable HCC in a global, multicenter, open-label, phase III trial (ClinicalTrials.gov number: NCT03434379) (64). Other anti-VEGF drugs are also being evaluated in the combined therapy with immunotherapy for HCC treatment (ClinicalTrials.gov number: NCT03170960 (Cabozantinib and Atezolizumab) and NCT03006926 (Lenvatinib and Pembrolizumab)).

### Inducing the Formation of Tertiary Lymphoid Structures

Although intra-tumoral infiltration by immune cells is a predictor of sensitivity to ICI treatment and many studies have focused on the role of T cell in antitumor responses, other immune cells have not been widely examined. Recently, studies revealed that the presence of intra-tumoral tertiary lymphoid structures (TLSs) improves ICI treatment of melanoma (141). TLSs are ectopic lymphoid aggregates that reflect lymphoid neogenesis occurring in non-lymphoid tissues in response to chronic inflammation, characterized by mature DCs in a T-cell zone adjacent to B-cell follicles including a germinal center (142, 143). TLSs are found in most types of cancer, with high TLS densities associated with improved clinical outcomes (144). In HCC, intra-tumoral TLSs are correlated with a decreased risk of early HCC recurrence after surgical resection, which may reflect ongoing, effective antitumor immunity (145). Therapeutic strategies to induce the formation of TLSs may enhance the antitumor immunotherapy of HCC (145). A reagent targeting LIGHT, a member of tumor necrosis factor superfamily of cytokines, can induce the formation of TLSs and can be combined with ICI to increase the number of TILs, conferring a survival benefit in mice with insulinomas (146). Other strategies aimed at stromal cells, which participate in the establishment of TLSs (147). Stromal cells derived from lymph nodes and induce TLSs cause infiltration of host immune cell subsets to suppress tumor growth *in vivo* (148).

### Locoregional Therapy

Locoregional therapies such as RFA can be as efficient as surgical resection of HCC nodules (149) but patients treated with this therapy frequently experience cancer recurrence. Although it is not effective as monotherapy, locoregional therapy causes tumor cell death *via* the release of tumor antigens and stimulation antitumor immunity (150), named as immunogenic cell death (ICD). ICD may enhance the anti-tumor immune reaction through the antigens and adjuvants released during this process. ICD of tumor cells results in the release of neoantigens which may be recognized by DCs followed by activation of the adaptive immune response (151). Moreover, heat shock proteins induced by RFA have been shown to enhance the immune response by activating the natural immune response and augmenting the antigen-specific cytotoxic T-cell response (152–154). Although the effect of immune activation by locoregional therapy alone is not sufficient for treating HCC, it may be an effective adjuvant for immunotherapy (150).

Tremelimumab combined with RFA or chemoablation for advanced HCC resulted a partial response in 26.3% of patients (5 of 19), with a clear increase in CD8^+^ T cells. Progression-free survival rates at 6 and 12 months were 57.1% and 33.1%, respectively, and the median overall survival was 12.3 months (65).

### Chemotherapy

Chemotherapeutic drugs alone for treating HCC, such as oxaliplatin, have shown limited effects on the overall survival of patients with advanced HCC. These cytotoxic drugs induce tumor cell death, which may also stimulate anti-tumor immunity by induced ICD. The cytotoxic effect also induces a decrease in the immunosuppressive cell population, such as MDSCs and T_reg_ cells (155, 156). High-dose chemotherapy, which is the proper strategy for the treatment of HCC, leads to the death of both tumor and immune cells. The suppressed immune system then loses its function and no longer targets therapy-resistant tumor cells.

Low dose metronomically administered chemotherapy can increase the ablation of immunosuppressive T_reg_ cells (156, 157), promote the maturation and activation of DCs (158, 159), and improve the activation and functionality of cytotoxic NK and CD8^+^ T cells (160). Treatment with metronomic cyclophosphamide affected gliomas by activating anti-tumor CD8^+^ T cell responses and immune memory in an immune-competent mouse model with implanted GL261 glioma (160). Pre-treatment with metronomic chemotherapy for HCC may enhance the effect of ICI and avoid unacceptable toxicity (161).

### Cytokines

Although cytokines have multiple functions in the formation of the immune system, cytokine treatment alone as an immunotherapy for HCC is limited. IFN-α was the first immunotherapy tested in many clinical trials. Although IFN-α has anti-proliferative, immunostimulatory, and anti-angiogenesis properties (162), most trials failed to show clinical benefits (163, 164).

Overexpression of cytokine CCL5 in CTNNB1-mutant HCC cells led to the recruitment of CD103^+^ DCs and antigen-specific CD8^+^ T cells, which may enhance the clinical outcome of ICI therapy (165). CCR4 expressed by T_reg_ cells can suppress anti-tumor immune response. In a phase I study, the safety and efficacy of combined mogamulizumab (anti-CCR4 antibody) and nivolumab are evaluated for patients with HCC, with four (27%) tumor responses among 15 patients. During treatment, the immune system activated with population of T_reg_ cells decreased and effector CD8^+^ cells increased (66). Although immunotherapy using cytokines alone is limited for treating HCC, the potential advantage of cytokines as adjuvants to enhance the clinical efficacy of immunotherapy is promising.

## Conclusion

Immunotherapy as monotherapy or combined with other therapeutic strategies has demonstrated clinical efficacy. Although some patients benefit from these therapeutic approaches, most patients suffering from advanced HCC do not. Novel immunotherapy strategies are currently being evaluated.

## Author Contributions

Conceptualization, XF and HL. Resources, CZ. Writing – Original Draft Preparation, CZ and YL. Writing – Review and Editing, CZ and SJ. Visualization, GC and DL. Supervision, JY, XF and HL. Funding Acquisition, HL. All authors contributed to the article and approved the submitted version.

## Funding

We are greatly indebted to the subjects enrolled in our study. This work was supported by the National Key Research and Development Program (2016YFC0906400), National Natural Science Foundation of China (81400656, 81872297, and 81874059), Zhejiang province analysis and test technology project (2018C37062), the Fundamental Research Funds for the Central Universities(2016XZZX002-05).

## Conflict of Interest

The authors declare that the research was conducted in the absence of any commercial or financial relationships that could be construed as a potential conflict of interest.
